# High Pressure Experimental Studies on CuO: Indication of Re-entrant Multiferroicity at Room Temperature

**DOI:** 10.1038/srep31610

**Published:** 2016-08-17

**Authors:** Rajesh Jana, Pinku Saha, Vivek Pareek, Abhisek Basu, Sutanu Kapri, Sayan Bhattacharyya, Goutam Dev Mukherjee

**Affiliations:** 1 Department of Physical Sciences, Indian Institute of Science Education and Research Kolkata, Mohanpur Campus, Mohanpur 741246, Nadia, West Bengal, India; 2 Department of Chemical Sciences, Indian Institute of Science Education and Research Kolkata, Mohanpur Campus, Mohanpur 741246, Nadia, West Bengal, India

## Abstract

We have carried out detailed experimental investigations on polycrystalline CuO using dielectric constant, dc resistance, Raman spectroscopy and X-ray diffraction measurements at high pressures. Observation of anomalous changes both in dielectric constant and dielectric loss in the pressure range 3.7–4.4 GPa and reversal of piezoelectric current with reversal of poling field direction indicate to a change in ferroelectric order in CuO at high pressures. A sudden jump in Raman integrated intensity of *A*_*g*_ mode at 3.4 GPa and observation of Curie-Weiss type behaviour in dielectric constant below 3.7 GPa lends credibility to above ferroelectric transition. A slope change in the linear behaviour of the *A*_*g*_ mode and a minimum in the FWHM of the same indicate indirectly to a change in magnetic ordering. Since all the previous studies show a strong spin-lattice interaction in CuO, observed change in ferroic behaviour at high pressures can be related to a reentrant multiferroic ordering in the range 3.4 to 4.4 GPa, much earlier than predicted by theoretical studies. We argue that enhancement of spin frustration due to anisotropic compression that leads to change in internal lattice strain brings the multiferroic ordering to room temperature at high pressures.

Multiferroic materials have attracted the imagination of scientific community for the novel magneto-electrical interactions and important applications in technological field. Even though many multiferroic materials have been discovered, a room temperature multiferroic system remained elusive, in which, ferroelectric order can be influenced with a change in magnetic order.In recent years cupric oxide (CuO) has generated a renewed interest in the scientific community as it holds a promise to be the room temperature type-II multiferroic with large polarization. CuO has attracted special interest due to discovery of high temperature superconductivity in cuprates and other wide applications in the industrial field, such as, fabrication of solar cells^1^,^2^; lithium ion batteries^3^; magnetic storage media, gas sensors^4^ etc. CuO is found to be quasi one dimensional (1D) antiferromagnet with a high Neel temperature (*T*_*N*_) of 230 K due to its large antiferromagnetic exchange interaction along zigzag *Cu*–*O*–*Cu* chains along [1 0 –1] direction^5^,^6^,^7^. Kimura *et al*.^8^ showed that CuO behaves as a type-II multiferroic in a short temperature range of 213 *K* < *T* < 230 *K* with both *T*_*C*_ and *T*_*N*_ coinciding at 230 K. The multiferroic behaviour in CuO is predicted to originate from spiral spin structure along [1 0 –1] direction due to magnetic frustration that breaks the inversion symmetry activating Dzyaloshinskii - Moriya interaction^8^,^9^,^10^,^11^. It presented the scientific community with a prototype simple bi-elemental compound that showed a promise to be a room temperature multiferroic. Therefore pressure seemed to be the other physical parameter that can be applied to test the possibility of stabilizing CuO as a type-II multiferroic at room temperature. High pressure neutron diffraction studies up to 1.8 GPa showed that *T*_*N*_ increases to 235 *K*^12^. Recent theoretical studies have shown that the antiferromagnetic super exchange parameter in CuO increases with pressure and in the pressure range 20–40 GPa it is predicted to exist in the multiferroic state at room temperature^13^. Interestingly high pressure X-ray diffraction and Raman spectroscopy measurements up to about 47 GPa have shown nanocrystalline CuO remains stable in its monoclinic (space group *C*2/*c*) state up to about 47 GPa^14^.

In the present work we have carried out a detailed experimental study on polycrystalline CuO using dielectric constant, dc resistance, Raman Spectroscopy, and X-ray diffraction measurements. We propose that multiferroic phase is induced in bulk CuO in the pressure range about 3.4–4.4 GPa at room temperature, much earlier than predicted from theoretical studies.

We have carried out dielectric constant (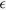) and dielectric loss (*δ*) measurements in the pressure range 0.08–9.9 GPa and in the frequency range 33 Hz–100 KHz in each pressure value. At low pressures and low frequencies, 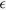 is calculated to be in the order of 10^4^. Such high value of 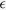 can be attributed to the presence of small amount of Cu^3+^ ions in the powder sample^15^,^16^. In addition to this Maxwell-Wagner effects arising due to sample-electrode interface and/or grain boundary effects in the polycrystalline materials also contribute to such large values of dielectric constant^17^,^18^. Figure 1(a) shows the frequency dependence of dielectric constant at different pressures. Few important observations are: (i) at all pressures 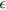 shows an exponential-like decrease;(ii) an increase in 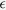 up to about 3.7 GPa pressure and then above 5 GPa pressure 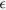 values drop suddenly and can not be distinguished in the figure. Therefore in Fig. 1(b), we have shown the frequency behaviour of 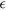 above 5 GPa. Interestingly general frequency dependence remains the same, but values seem not to change much with pressure. Therefore to understand the effect of extrinsic and intrinsic contributions to the obtained dielectric constant, we have plotted the representative frequency dependence of 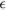 and dielectric loss at about 8 GPa pressure (Fig. 1(c)). The loss show a peak at about 16 KHz frequency that coincides with the drop in the dielectric constant indicating a Debye-like relaxation behaviour arising from Maxwell-Wagner effect. The strong frequency dependence in dielectric constant can be modeled in terms an equivalent circuit consisting of two parallel R-C circuits connected in series, where one of the capacitance comes from the extrinsic effects and other corresponds to bulk intrinsic effects. At high frequencies, above the peak frequency of the dielectric loss, the capacitance arising due to extrinsic effects gets shorted and intrinsic bulk behaviour is observed^17^,^18^,^19^.

Figure 2(a) shows the pressure dependence of dielectric constant and dielectric loss (*δ*), at a frequency of 100 KHz. At the lowest pressure of our study, 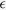 is estimated to be 400 at a frequency of 100 KHz. Such high value of 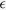 can be attributed to the presence of small amount of Cu^3+^ ions in the powder sample^15^,^16^. As pressure increases, 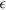
(*P*) remains almost constant up to about 1 GPa and then rapidly rises up to 3.7 GPa, followed by a sharp drop to a value of about 5. *δ*(*P*) shows an anomalous behaviour in the range 3.7 to 4.7 GPa with a sharp peak at 4.4 GPa. In Fig. 2(b) 1/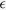
(*P*) is found to be linear in the range 1–3.4 GPa indicating a Curie-Weiss type behaviour. All the above features in the dielectric property of CuO are indicative of ferroelectric transition. Re-entrant ferroelectric behaviour has been observed in *PbTiO*_3_ systems at very high pressures, which is attributed to short range coulomb interactions and is preceded by structural transitions^20^,^21^. We have carried out 4-probe dc resistance measurement of the sample using Keithly 2400 current source and Keithley 2010 digital voltmeter. The variation of dc resistance (*R*_*dc*_(*P*)) with pressure is shown in Fig. 2(c). At low pressures the resistance is found to be of the order of 13 MΩ indicating the insulating nature of the sample. It can be seen in the figure that dc resistance drops by almost three orders of magnitude to a value of 150 KΩ in the range about 3 to 4.5 GPa and then goes back to the mega-Ohm range. Similar drop in resistance more than two orders of magnitude below 230 K has been observed in polycrystalline CuO, that coincides with the antiferromagnetic transition in the sample^22^. Electron diffraction experiments at ambient temperature have shown the presence of quasi 1D zigzag charge stripes in p-type CuO^23^ indicating the presence of strong spin-charge coupling in the system. Therefore one of the explanation for sudden decrease in resistance in CuO above 3 GPa may be attributed to the movement of trapped charges under the application of external electric field and that coincides with the onset of change in polarization in the sample. Above 4 GPa, complete onset of long-range antiferromagnetic order can pin the charges and stop their movement. This probably results in sudden increase of resistance above 4.5 GPa.

To confirm whether the ferroelectric order is induced by pressure, we attempted to measure the piezoelectric current in our sample by following the method prescribed by Kimura *et al*.^8^ for measuring the pyroelectric current. First the sample was clamped at an initial pressure of about 0.08 GPa. Then to pole the sample a positive dc electric field of value, *E* = 2 KV/cm was applied. Next pressure was slowly increased to about 8 GPa and the field was kept on for some time. After the poling procedure, the electric field was switched off. The electrical leads on the opposite faces of the sample were kept shorted for an hour. Then current was measured by slowly reducing the pressure. The same procedure was followed again by reversing the applied electric field. However, unlike in the case of pyroeletric current measurement with respect to temperature, in the present case we could not carry out a scan of pressure during pressure decrease. The pressure during scanning at the sample was not reproducible as: (i) while the hydraulic rams re-treated it was difficult to read the pressure value at the sample, (ii) since the sample was in bulk form, it takes some time to adjust to new pressure value. Therefore, we noted down our current measurements after waiting for 5 minutes at each step of pressure release to make sure the sample-pressure stabilized for a meaningful pressure measurement as the interest was to see whether the current changes its direction after poling reversal. The result is shown in Fig. 2(d). There are few interesting observations: (i) current is found to be in the nano-ampere range; (ii) sign of current is reversed after the reversal of poling field; (iii) current drops to a low value below a pressure of about 5 GPa (for both the poling directions). The above effects can be due to the piezoelectric property of the sample and reversal of current with reversing the poling field shows that the polarization seems to change with the direction of the poling field. It indicates to the ferroelectric behaviour of CuO above about 5 GPa pressure as ferroelectric ceramics can be made piezoelectrically active by the application of poling field.

Selective Raman spectra at different pressures are shown in Fig. 3, where no structural transition is observed. Group analysis decomposition of normal modes of vibration at zone centre has given only three (*A*_*g*_ + 2*B*_*g*_) Raman active modes^24^. Our ambient Raman spectrum contains three peaks at 292, 342 and 625 cm^−1^ in close agreement with earlier reported values^25^,^26^. The most intense peak at 292 cm^−1^ is assigned to *A*_*g*_ mode and other two less intense peaks are assigned to *B*_*g*_ modes^26^. For analysis, Raman spectra are normalized with respect to the Bose Einstein thermal factor by dividing the raw spectra by the factor (*n*(*ω*) + 1), where *n*(*ω*) = 1/(*exp*(−*E*/*k*_*B*_*T*)−1); *E* is the energy of the mode, *k*_*B*_ is the Boltzman constant and *T* is the room temperature value. All the modes are fitted to the standard Lorentzian function. A representative plot is shown in Figure 1 in the Supplementary Information. *A*_*g*_ mode is our point of interest as it involves the movement of O atom along *b*-axis and hence will be most susceptible to the ferroelectric transition as the ferroelectric behaviour is found along the *b*-axis in CuO^27^. Different fitting parameters of the *A*_*g*_ mode show several interesting anomalous changes. Frequency of *A*_*g*_ mode increases linearly with pressure, however there is a definite change in slope at about 3.4 GPa (Fig. 4(a)). The slope decreases from 4.8(4) cm^−1^/GPa below 3 GPa to 2.4(1) cm^−1^/GPa above 3 GPa. In the absence of any structural transitions such a small change of slope in *A*_*g*_ mode can be attributed to small change in Grüneisen parameter arising from its electronic contribution. The full width half maximum (FWHM) of the *A*_*g*_ mode decreases rapidly till about 3.4 GPa and then increases with pressure (Fig. 4(b)). The FWHM of a Raman mode is related to the lifetime of the phonon and it may get affected due to coupling of phonons to electrons or their spins. Since there is no indication of a structural transition, the observed minimum in the FWHM of the *A*_*g*_ mode at about 3.4 GPa can only be related to an electronic transition coming from spin-phonon coupling process. In Fig. 4(c) we have plotted the normalized integrated intensity of the *A*_*g*_ mode with respect to pressure, which shows an anomalous jump at 3.4 GPa. Raman scattering intensity is directly proportional to the square of mode polarizability. Therefore the sudden increase in the intensity can be attributed to change in polarization of the Cu-O-Cu bonding line due to strong dynamic O-ion displacements.

As a complementary study to Raman measurements we have conducted careful *in situ* X-ray diffraction measurements under pressure up to about 43 GPa. Over the whole pressure range, no remarkable changes in the diffraction patterns are observed, consistent with the earlier studies^14^ (Supplementary Figure 2). Indexing of the collected XRD patterns at various pressure are carried out followed by structural analysis taking the atom positions from high pressure neutron diffraction studies^5^,^28^. A representative Riteveld refinement of the ambient XRD pattern is shown in the Figure 3 in the Supplementary Information. Since the previous anomalies are observed around 3.4 GPa, in the present case we have restricted analysis of our XRD patterns below 10 GPa. In Fig. 5(a) we have shown pressure variation of relative change in lattice parameters *a*, *b*, *c*, with respect to the ambient pressure value. Interestingly the compression behaviour of the unit cell is found to be anisotropic with *a*-axis expanding with pressure and also one can see that relative change in volume almost scales with the relative change in *b*-axis up to about 6 GPa. To see whether the electronic transitions affect the internal strain we have plotted reduced pressure *H* 
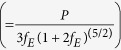
 with respect to the Eulerian strain *f*_*E*_ 
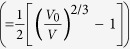
29. The behaviour should be linear and is given by the equation^30^,^31^,


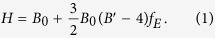


However in Fig. 5(b) one can clearly see a kink at about 3.4 GPa even in the absence of any structural transition. Linear fit of the data points below 3.4 GPa gives *B*_0_ = 103.4(6) GPa and *B*′ = 5.3 (2) and above 3.4 GPa gives *B*_0_ = 105.5(5) GPa and *B*′ = 4.2(2). Similar anomalies have been observed in many different systems due to electronic topological transitions^29^,^32^. Increase in Cu-O-Cu bond angle makes the antiferromagnetic super-exchange parameter stronger and can increase *T*_*N*_ to room temperature^12^,^33^. In Fig. 5(c) it can be seen that with increase in pressure the (2,0,−2) Bragg peak is found to shift to lower 2Θ values indicating a lattice expansion perpendicular to (2,0,−2) plane, that shows that the Cu-O-Cu bond angle increases along [1 0 –1] direction with pressure.

XRD analysis shows anisotropic compression in the unit cell with an expansion along *a*-axis and a maximum compression along *b*-axis. This may result in increase of spin frustration because of the competition of antiferromagnetic and ferromagnetic interactions along [1 0 –1] and [1 0 1] direction respectively. In one possibility as discussed in many reports, above spin frustration may result in breaking of symmetry due to DM interactions and the ferroelectric order emerges with its polarization vector along *b*-axis. However breaking of symmetry can lead to additional Raman bands. In low temperature Raman and IR spectroscopy studies many extra low intensity modes are observed^34^,^35^, which are attributed to the strong spin-phonon interaction and lattice instability even in the incommensurate antiferromagnetic state of CuO. In the present case we do not see any extra Raman bands. The reason may be since these are very low intensity modes, they are masked by back ground intensity coming from room temperature photons or averaging effect due to powder samples. In the present work we observe a strong electron-phonon interaction from the observed minimum in the FWHM of *A*_*g*_ mode and the change in lattice internal strain at 3.4 GPa. This is also reflected in the decrease in slope in *A*_*g*_ mode pressure behaviour at 3.4 GPa. *A*_*g*_ mode is attributed to O-atom vibrations along *b*-axis and can be modeled in terms of a 1D diatomic chain of Cu and O atoms. The energy of the phonon at Brillouin Zone (BZ) boundary can be given by, (2 *K*/*M*_*O*_)^1/2^, where *K* is the force constant and *M*_*O*_ is the mass of the oxygen atom. Small mass of O gives rise to large vibrational amplitudes that may favour large magnetic fluctuations. Since the Debye temperature of CuO is much larger than room temperature(~560) K^36^, the amplitude of the vibrational mode (*u*) can be expressed as (under harmonic approximation), 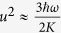
37,38. At the onset of magnetic transition large critical fluctuations will reduce the force constant *K* and will act as negative feedback for frequency of vibration at BZ boundary. Therefore the phonon frequency seems softer with respect to the paramagnetic phase and hence the slope changes. Even though from the present experiments we can not definitely comment on the magnetic state of the CuO sample above 4 GPa, the presence of strong electron-spin interaction as evident from literature indicates to a magnetic transition induced by the observed ferroic transition. An increase in the antiferromagnetic superexchange parameter is observed from the high frequency cutoff in Raman spectra due to spinon contribution as shown in the Figure 4 in the Supplementary Information. The transition of the sample to a high resistive state indicating an insulating nature, an antiferromagnetic order is more probable. The importance of magnetic disorder due to non-linear behaviour of the lattice is apparent in the realization of room temperature binary type II multiferroic from the present experimental study. However a more detailed magnetic study is required at this pressure to strongly defend our conclusion. Therefore we feel that our results will induce more research activity for accurate experimental and theoretical modeling of the multiferroic systems at high pressures.

In summary, we have carried out detailed investigations on polycrystalline CuO at high pressures using both electrical and structural experimental techniques. Our high pressure dielectric constant and Raman spectroscopy measurements show a paraelectric to ferroelectric transition in the sample in the pressure range about 3.4–4.4 GPa. A distinct change in the slope and the FWHM of the *A*_*g*_ Raman mode indirectly indicate to a change in the magnetic order of the sample. The slight mismatch in the experimental transition pressures between the electrical and structural measurements can be related to the different types of pressure cells used for the study. Our results show that CuO can exist as a type II multiferroic at a pressure as low as 4.4 GPa at room temperature. Our result suggests that one can look for a synthesis protocol at high pressures to stabilize CuO in the multiferroic state at room temperature.

## Method

CuO is synthesized in laboratory using a typical synthesis protocol using two aqueous solutions of *CuCl*_2_.2*H*_2_*O* and *NaOH* prepared with distilled water. The hydrolyzed product is centrifuged followed by washing and repeated heating at 1073 K to improve the phase quality. The final product is characterized for phase purity by X-ray diffraction (XRD) measurements. All the observed XRD peaks are indexed to monoclinic structure with space group *C*2/*c* and unit cell parameters: *a*_0_ = 4.6946(3), *B*_0_ = 3.4182(2), *c*_0_ = 5.1329(3) Å, *V*_0_ = 81.202(7) Å^3^, *β*_0_ = 99.65(4)^0^, which are in good agreement with the literature^14^. High pressure dielectric constant (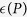), dielectric loss (*δ*(*P*)) and electrical resistance (*R*(*P*)) measurements are carried out with a GW Instek make Precision LCR Meter, Model–800G using a Toroid anvil (TA) apparatus^39^,^40^ up to about 9 GPa. The TA apparatus is pressure calibrated using Bi *I*–*II* and Yb *hcp*–*bcc* transitions at 2.65 GPa and 4 GPa respectively. A pellet of the sample of 3 mm diameter and 1 mm thickness is sandwiched between two copper plates of thickness 0.1 mm and placed inside a Teflon cup with a lid. Teflon cup is inserted into the hole of diameter 5 mm drilled at the central part of the toroid shaped pyrophylite gasket. Two thin steel wires of 20 micron dia are attached to the copper plate and taken out through small holes in the Teflon cup and then through the side holes of the gasket. The gasket with whole sample assembly is compressed and locked between two toroid shaped opposed anvils by the 300 ton hydraulic press for 30 minutes at an initial pressure of 0.5 GPa to ensure a good contact between electrodes and the sample. The initial compression reduced the sample thickness to about 0.5 mm and no further reduction has been observed with increasing pressure up to 9 GPa and back to ambient condition. Also no deformation has been observed in the Teflon cup, which ensures a reproducible data in the pressure range of 0–9 GPa. AC resistance measurements are carried out in the frequency range 33 Hz −1 MHz. Short circuit trimming of LCR Meter is done to calibrate the contact probes for stray capacitance and series impedance of different metal contacts and steel wire. At each pressure 20 successive data are taken for the precision in measurements. Pressure dependent dielectric constant was calculated from the measured capacitance using the relation, 
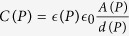
, where *A*(*P*) and *d*(*P*) are area of the copper plates and thickness of the sample respectively at pressure *P* and 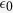 is the free space permittivity. Area of the copper plates can be assumed to be constant for pressures up to 9 GPa. For Raman spectroscopy and X-ray diffraction experiments, high pressure is generated using a diamond anvil cell (Easylab Co., UK) with diamond culets of size 300 *μ*m. Sample was loaded in a 100 *μ*m hole of preindented stainless steel gasket of thickness of about 45 *μ*m together with 4:1 methanol–ethanol mixture as liquid pressure transmitting medium. For Raman measurements a thin pellet of CuO of approximate dia 50–60 *μ*m and thickness 10–20 *μ*m is loaded in the pressure cell. Raman measurements at high pressures are carried out in a back scattering geometry with a resolution of 1.2 cm^−1^ using Horiba Jobin Yvon LabRam HR 800 Raman spectrometer and the sample was excited with the *Ar*^+^ ion (488 nm) laser at power 6 mW. Pressure is measured using ruby fluorescence technique^41^. High pressure x-ray diffraction measurements are performed at room temperature at XRD1 beamline in Elettra synchrotron source in Italy at an wavelength of 0.689 Å. Fine silver powder is mixed with the sample that acted as pressure marker^42^. Collected XRD patterns are indexed using freely available software Dicvol^43^ and then analyzed using the Rietveld refinement program GSAS^44^,^45^.

## Additional Information

**How to cite this article**: Jana, R. *et al*. High Pressure Experimental Studies on CuO: Indication of Re-entrant Multiferroicity at Room Temperature. *Sci. Rep.* **6**, 31610; doi: 10.1038/srep31610 (2016).

## Supplementary Material

Supplementary Information

## Figures and Tables

**Figure 1:**
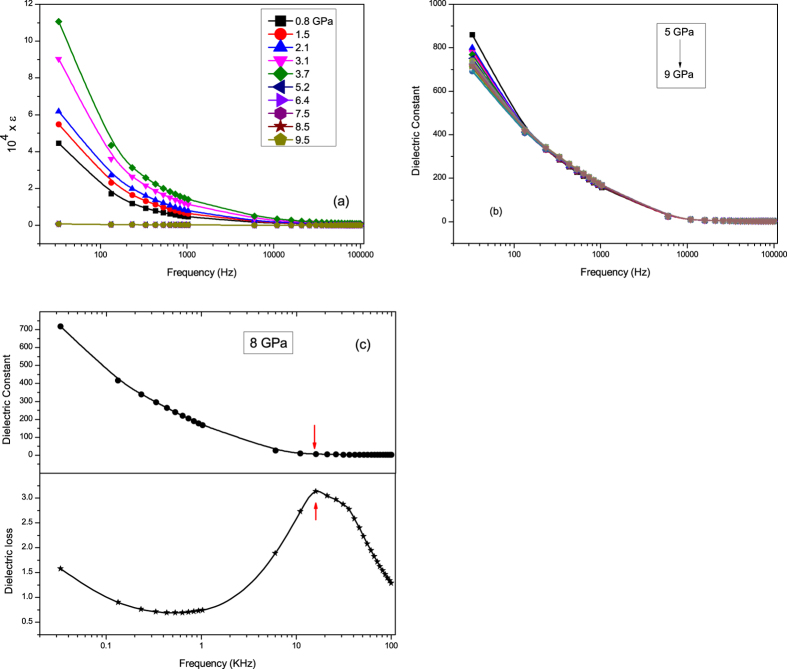
(**a**) Frequency dependence of dielectric constant at selected pressures of CuO. (**b**) Frequency evolution of dielectric constant above 5 GPa pressure. At all pressures the dielectric behaviour seem not change much compared to the values below 3.7 GPa. (**c**) Frequency dependence of dielectric constant and loss at 8 GPa pressure showing Debye-like relaxation behaviour at high frequencies.

**Figure 2:**
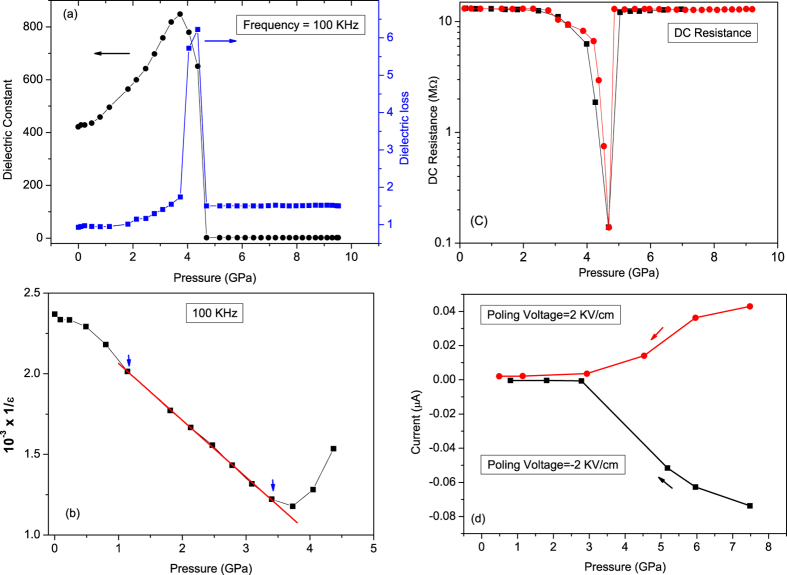
(**a**) Pressure evolution of dielectric constant (dots) and dielectric loss (squares) of CuO at 100 KHz. (**b**) Inverse of dielectric constant (dots) show linear behaviour with pressure in the range 1–3.4 GPa and can be fitted to a straight line (red line) exhibiting Curi-Weiss type behaviour in the paraelectric region. The data are fitted to Curie-Weiss equation: 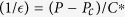, where *P*_*c*_ (the critical pressure of transition) and C* (the equivalent Curie constant in pressure domain for CuO) are the parameters. From the fit *P*_*c*_ can be estimated to a value of 6.8 GPa. (**c**) Pressure dependence of dc resistance from two different experiments show a decrease of three orders of magnitude in the pressure range 3.0–4.5 GPa followed by a sudden increase. (**d**) Measured piezoelectric current under a poling voltage of (+/−) 2 KV/cm. The piezoelectric current changes its sign depending on the poling voltage direction showing change in direction of polarization. Also below 5 GPa, the measured current drops to a low value due to loss of ferroelectric order.

**Figure 3:**
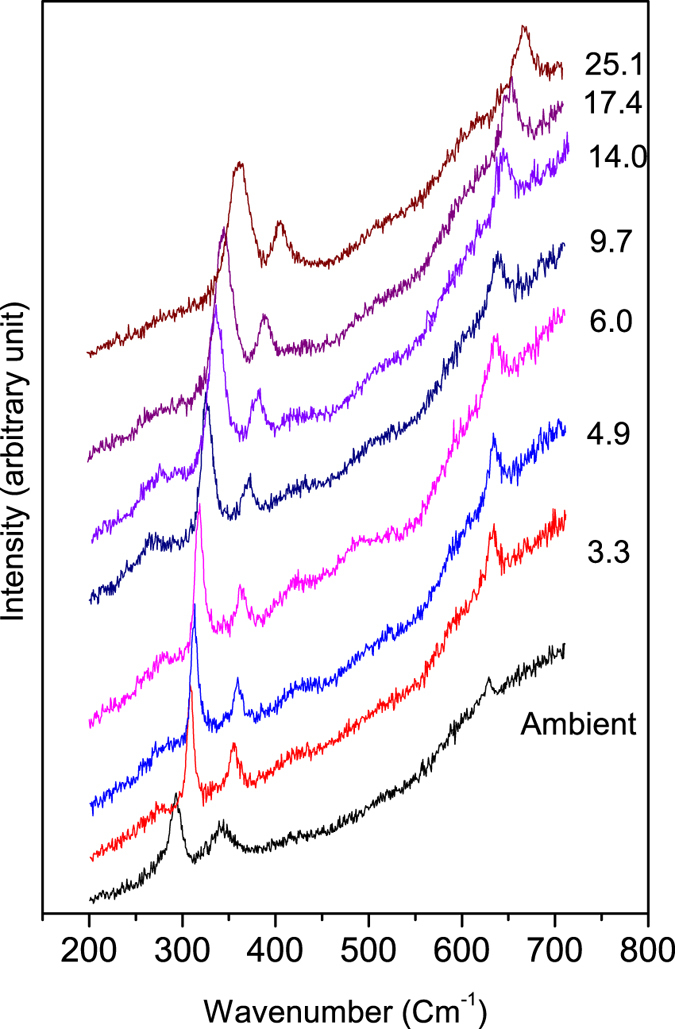
Selected Raman spectra of CuO at various pressures.

**Figure 4:**
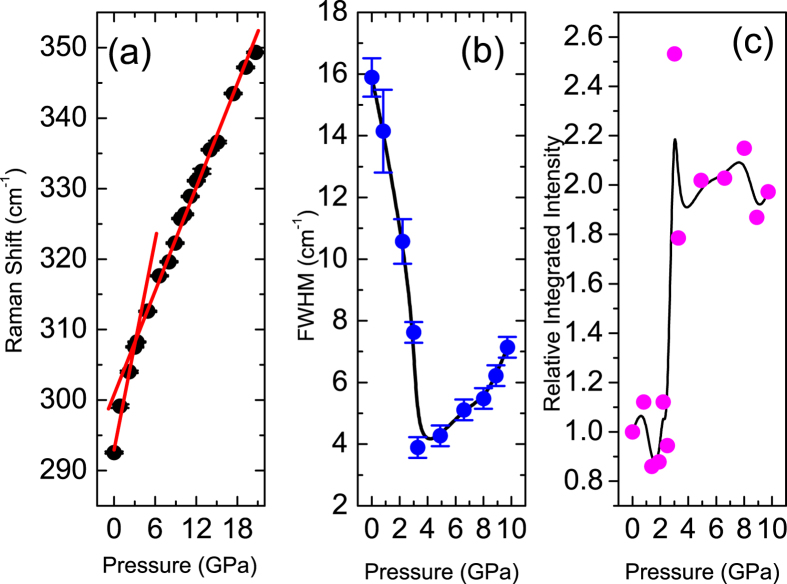
(**a**) Linear pressure evolution of *A*_*g*_ Raman mode with a slope change at 3.4 GPa; (**b**) FWHM of *A*_*g*_ Raman mode showing a minimum at 3.4 GPa; and (**c**) relative integrated intensity of the *A*_*g*_ Raman mode with respect to the ambient pressure value show an abrupt jump at 3.4 GPa indicating a change in polarization of the above said mode.

**Figure 5:**
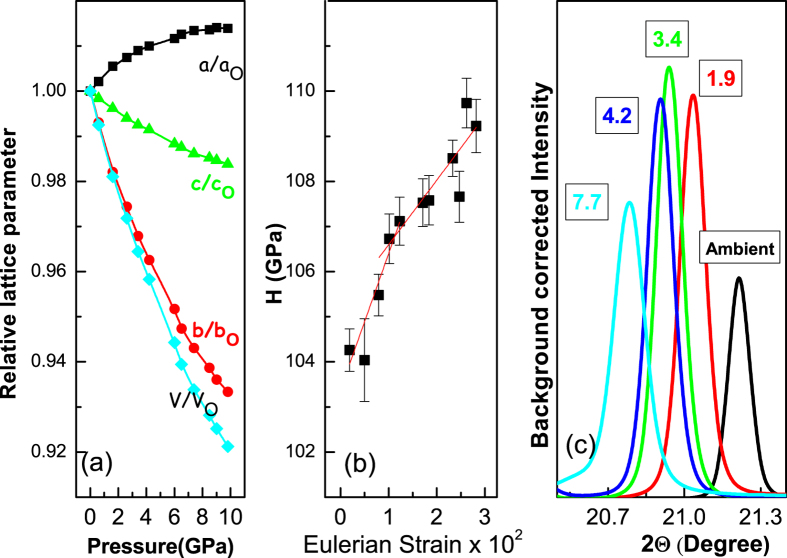
(**a**) Pressure evolution of relative lattice parameters with respect to the ambient pressure value up to 10 GPa. The monoclinic axis almost follows the unit cell volume compression indicating its importance in enhancement of magnetic frustration. (**b**) Plot of normalized pressure *H versus f*_*E*_ in the monoclinic phase of CuO, which shows a kink at 3.4 GPa. Red line shows the linear fit of the data above and below 3.4 GPa, separately. (**c**) Background subtracted XRD pattern of CuO at various pressures are plotted highlighting the change in position of (2,0,−2) Bragg peak indicating an expansion perpendicular to this plane.
