# Supplementary material for: High Pressure Experimental Studies on CuO: Indication of Re-entrant Multiferroicity at Room Temperature
Source: Sci Rep. 2016 Aug 17;6:31610. doi: 10.1038/srep31610 (PMC4987678; doi:10.1038/srep31610)
Supplement: Supplementary Information [file srep31610-s1.pdf]

High Pressure Experimental Studies on CuO: Indication of Re-entrant Multiferroicity at Room Temperature

Rajesh Jana, Pinku Saha, Vivek Pareek, Abhisek Basu,* Sutanu Kapri[1], Sayan Bhattacharyya[1], and Goutam Dev Mukherjee†

Department of Physical Sciences, [1]Department of Chemical Sciences,

Indian Institute of Science Education and Research Kolkata,

Mohanpur Campus, Mohanpur 741246, Nadia, West Bengal, India.

(Dated: July 14, 2016)

* Present Address: Geophysical Laboratory, Carnegie Institution of Washington, Washington, DC 20015-1305, USA

† Corresponding Author: goutamdev@iiserkol.ac.in

Supplementary Information

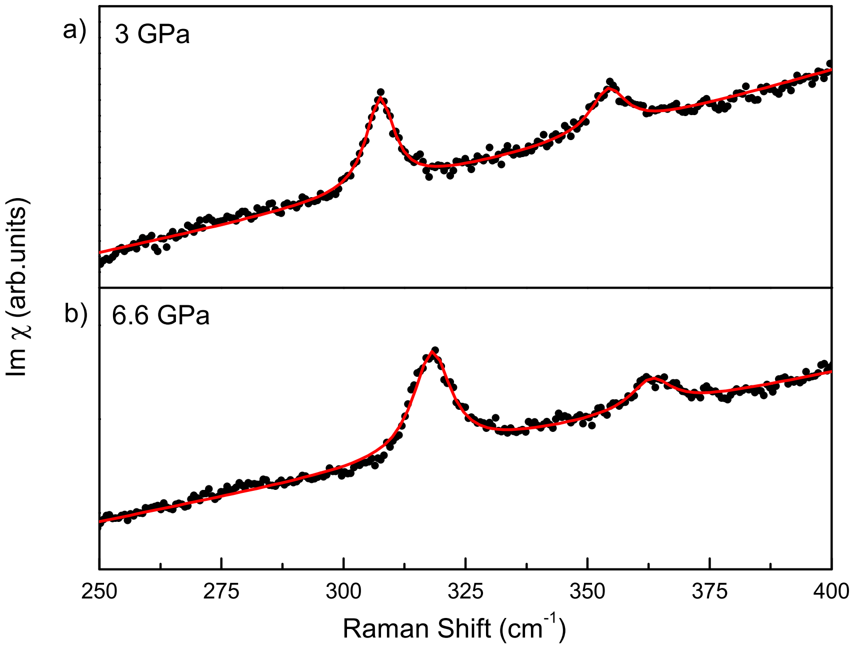

Figure 1. Intensity of Raman spectra normalized with respect to Bose Einstein thermal factor and then fitted to Lorentzian function at two pressures.

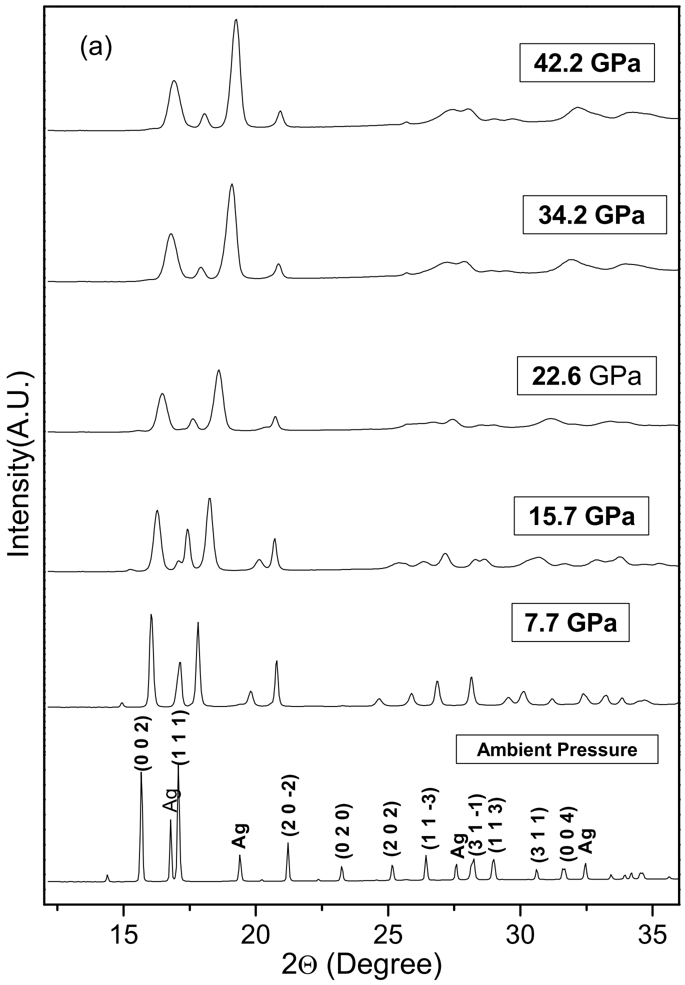

Figure 2. Pressure evolution of XRD pattern of CuO upto 42 GPa, the highest pressure of this study. The XRD patterns show a systematic compression in volume without any structural transition.

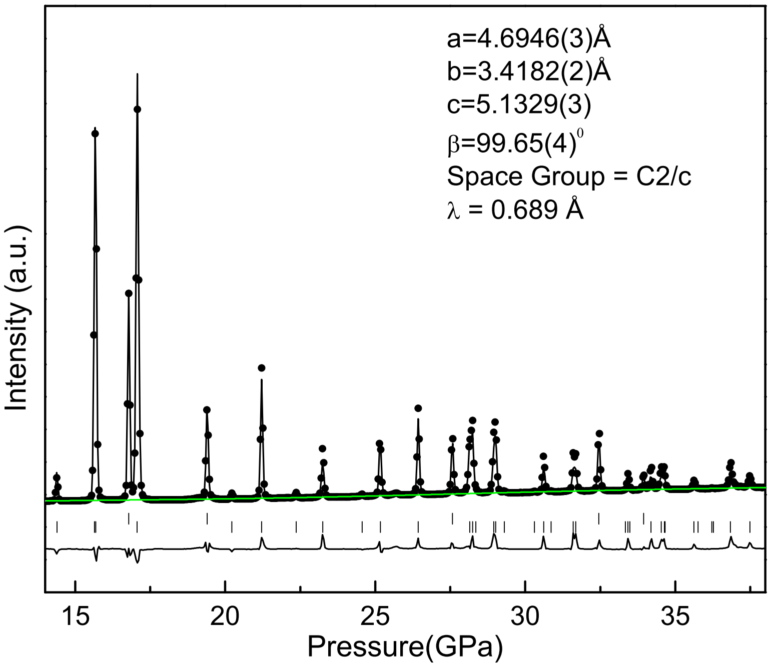

Figure 3. Profile fit of the XRD pattern of CuO at ambient condition using the indexed lattice parameters. Line though the data points show the fit to the data. Lower line-curve shows the difference between the fitted and observed pattern. Horizontal lines show the Bragg peaks of CuO and Ag pressure marker (slightly shifted up).

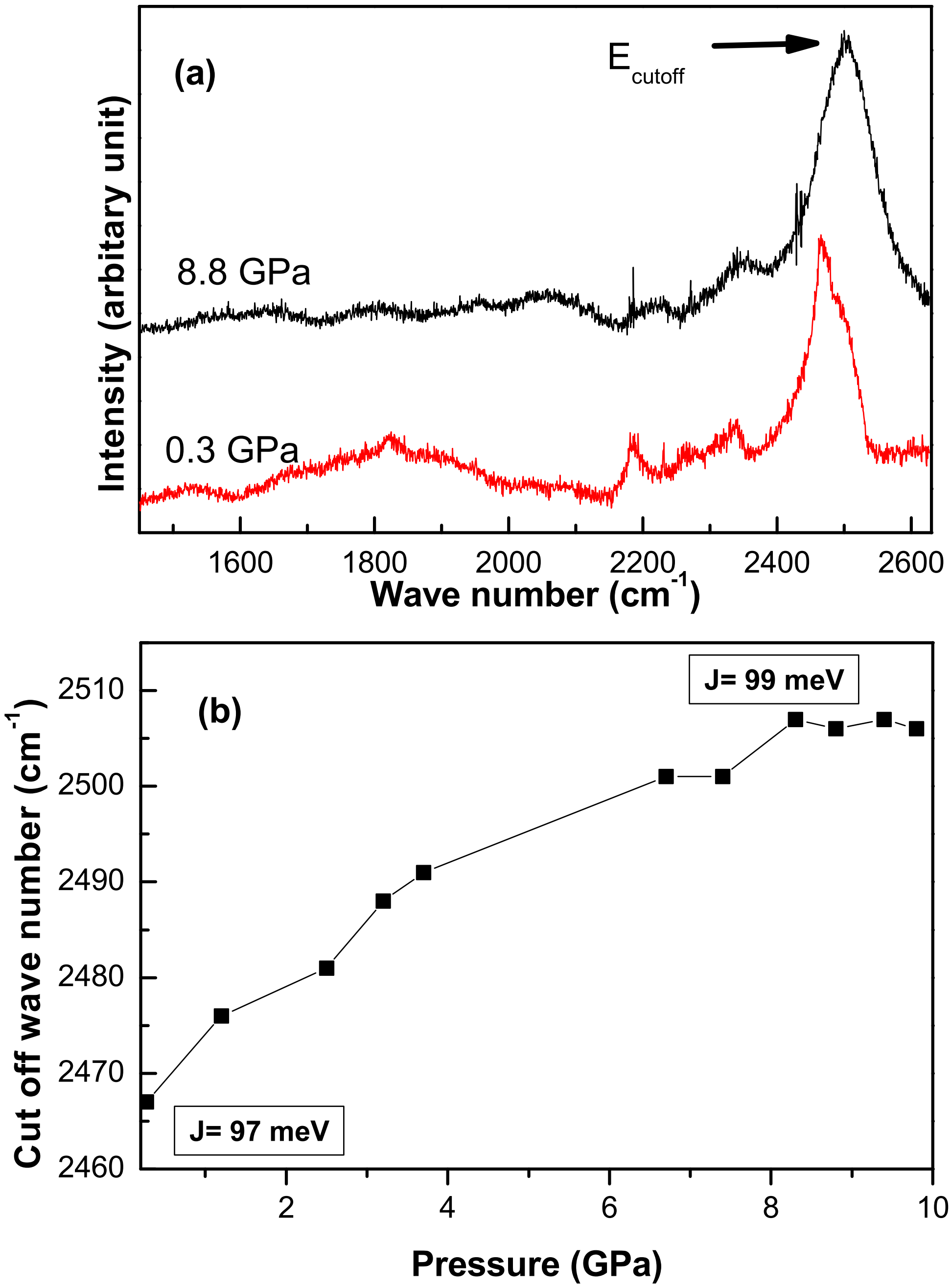

Figure 4. (a) Raman spectra of CuO at two pressures in the range 1400 - 2600 cm^{-1}. Both the spectra show sharp fall after a sharp peak probably due to sharp cutoff (E_{cutoff}) arising from the spinon contribution to Raman spectra(Ref.27). (b) The E_{cutoff} value increases till about 6.7 GPa and then almost stabilizes. Calculation of antiferromagnetic superexchange parameter (J) show it to be in the range 97 - 99 meV that matches well with the value reported in literature.
